# Genetic Networks Lead and Follow Tumor Development: MicroRNA Regulation of Cell Cycle and Apoptosis in the p53 Pathways

**DOI:** 10.1155/2014/749724

**Published:** 2014-09-11

**Authors:** Kurataka Otsuka, Takahiro Ochiya

**Affiliations:** ^1^Division of Molecular and Cellular Medicine, National Cancer Center Research Institute, 5-1-1 Tsukiji, Chuo-ku, Tokyo 104-0045, Japan; ^2^Division of Research and Development, Kewpie Corporation, Sengawa-cho, Chofu-shi, Tokyo 182-0002, Japan

## Abstract

During the past ten years, microRNAs (miRNAs) have been shown to play a more significant role in the formation and progression of cancer diseases than previously thought. With an increase in reports about the dysregulation of miRNAs in diverse tumor types, it becomes more obvious that classic tumor-suppressive molecules enter deep into the world of miRNAs. Recently, it has been demonstrated that a typical tumor suppressor p53, known as the guardian of the genome, regulates some kinds of miRNAs to contribute to tumor suppression by the induction of cell-cycle arrest and apoptosis. Meanwhile, miRNAs directly/indirectly control the expression level and activity of p53 to fine-tune its functions or to render p53 inactive, indicating that the interplay between p53 and miRNA is overly complicated. The findings, along with current studies, will underline the continuing importance of understanding this interlocking control system for future therapeutic strategies in cancer treatment and prevention.

## 1. Introduction

Cancer is commonly an age-related disease triggered by the accumulation of genomic mutations that lead to the dysregulation of tumor-suppressive genes and/or protooncogenes. For example, the functions of* TP53* (tumor-suppressive gene) and c-*MYC* (oncogene) have been extensively investigated, and their critical roles in complexly regulating tumorigenesis, including cell-cycle progression/arrest, apoptosis, senescence, and energy metabolism, have been uncovered [1–4]. Specifically, the significance of tumor suppressor p53 has been suggested by the fact that DNA mutation or loss of* TP53* is observed in many types (over 50%) of human tumors and by the possibility that the dysfunctions affect the p53 signaling network in over 80% of tumors [5, 6]. As a transcriptional activator, the p53 protein induces various kinds of tumor-suppressive genes, such as* p21* (G_1_/S-arrest),* 14-3-3σ* (G_2_/M-arrest), and* PUMA* (apoptosis) [7–10]. p53 has also been reported to negatively regulate specific proteins: for instance, the p53-mediated repression of the cell-cycle regulators, such as cyclin-dependent kinase 4 (CDK4) and cyclin E2, may lead to cell-cycle arrest [10, 11]. These prove the pivotal roles of p53 as a cellular gatekeeper.

Recently, it has been realized that small noncoding RNAs known as microRNAs (miRNAs) contribute to many human diseases, including cancers; that a general downregulation of miRNAs is observed in cancers as compared with normal tissues; and that miRNA expression profiles can be used to classify poorly differentiated tumors [12]. In addition, some kinds of miRNAs are shown to be connected to a well-studied tumor-suppressive or oncogenic network [13]. It remains to be investigated how miRNAs are regulated by transcription factors, but it is suggested that p53 enters the miRNA world to control the expression patterns of some miRNAs and promote cell-cycle arrest and apoptosis through the miRNA effector pathway.* miR-34a* is one of the representative miRNAs under the direct control of p53, and this upregulation induces cell-cycle arrest and apoptosis [14–18]. Moreover, there are many studies about miRNA effects on cell proliferation and survival in cancers, with attention given to the interplay between p53 and the miRNA network. In this review, we will focus on the regulation of the cancer cell cycle and apoptosis by miRNA linked with the p53 axis. We will also summarize the key miRNAs concerned with the cell cycle and apoptosis in cancers.

## 2. miRNA Discovery, Biogenesis, and Mechanism

The first miRNAs discovered were* lin-4* and* let-7*, both of which are the key regulators in the pathway controlling the timing of postembryonic development in* Caenorhabditis elegans* [19–21]. After this discovery, miRNAs have been identified in diverse organisms, such as worms, flies, mice, humans, and plants. Several miRNAs are conserved among different species, indicating that these miRNAs might have important functions and modulate gene expression. Currently, in humans, over 2,000 microRNAs have been identified or predicted based on the miRBase database (http://www.mirbase.org/). Computational analyses suggest that about 5,300 genes contain miRNA target sites: ~30% of human genes might be subject to the translational regulation of miRNAs [22, 23].

miRNAs are initially transcribed by RNA polymerase II/III into primary transcripts (pri-miRNAs) [24, 25], which are processed by the complex of RNase III enzyme, Drosha, and its partner DGCR8 [26]. The pri-miRNAs are converted into ~65 nucleotides (nt) of a stem-loop precursor (pre-miRNA) [27]. These pre-miRNAs are transported to cytoplasm by Exportin-5/Ran-GTP and processed by another RNase III, Dicer, to generate a double-strand RNA of about 19–25 nt in length [28–30]. One strand of miRNA gives rise to the mature miRNA, which is incorporated into the RNA-induced silencing complex (RISC). The miRNAs guide the RISC complex to the 3′-untranslated region (3′-UTR) of the target mRNAs, leading to the translational repression or destabilization of the mRNA [31, 32]. In animal systems, the recognition of target mRNA usually requires the “seed” sequence, which is 2–8 nt from the 5′-end of the miRNA [22, 33]. Unlike with plant systems, because of this imperfect complementarity, there are extensive base-pairings to the sequence of mRNAs, and this makes it more complicated to predict miRNA targets and study miRNA biology. Recently, it has been shown that animal miRNAs can induce the degradation of target mRNAs (mRNA degradation and decay) besides translational repression: inhibition of translation elongation; cotranslational protein degradation; competition for the cap structure; and inhibition of ribosomal subunit joining [34–37]. However, the exact order and impact of these events still need to be investigated further.

## 3. p53 Transactivation Function in a Relationship with Tumorigenesis

Based on numerous studies at both structural and functional levels, p53 is known as a key player in genome stability and tumor suppression. In an unstressed condition, the expression level of p53 is kept low by the activity of an E3 ubiquitin ligase, mouse double minute 2 (MDM2) [38–40]. Under stressed conditions, p53 is activated in response to diverse intrinsic and extrinsic signals, such as DNA damage, oncogene activation, and hypoxia. As a sequence-specific transcription factor, the activated p53 acts directly on cancer-associated pathways to suppress tumor progression by modulating cell-cycle arrest, senescence, apoptosis, angiogenesis, or invasion and metastasis [41–43]. There are also demonstrations showing that p53 is involved in the regulation of DNA repair, oxidative stress, energy metabolism, and differentiation [44–48]. The approach of genome-wide analyses has identified many p53-binding sites and p53-regulated genes which are related to tumorigenesis and various stress signals [49, 50]. Recent works have highlighted that p53 directly induces some specific miRNAs which function as tumor suppressors through a novel transcriptional mechanism. Now, although unknown aspects of the mechanism still need to be investigated, the cooperative contribution of p53 and miRNAs has been shown to be more important for tumor formation and development.

## 4. miRNA Network with p53: Cell Cycle and Apoptosis

### 4.1. *miR-34* Family

In 2007, several groups reported that the* miR-34* family members are direct p53 targets and that their expression level is strongly upregulated by genotoxic stress in a p53-dependent manner, inducing cell-cycle arrest and apoptosis [14–16, 51, 52]. In mammalians, the* miR-34* family is composed of* miR-34a*,* miR-34b*, and* miR-34c*, which are encoded by two different genes in the* miR-34a* and* miR-34-b/c* loci. With the overexpression of the* miR-34* family in certain kinds of cell lines, microarray analyses unveiled hundreds of putative candidate target genes of* miR-34s* [15, 16, 18]. Actually, ectopic expression of* miR-34s* promotes cell-cycle arrest in the G_1_ phase, senescence, and apoptosis by directly repressing CDK4, CDK6, cyclin E2, E2F3, MYC, and B-cell CLL/lymphoma 2 (BCL-2) [53]. Note that the triggering event of cell-cycle arrest or apoptosis by* miR-34s* depends on the cell type and context, and the expression level of* miR-34s* would affect the decision to proceed [15, 17, 54]. As seen in the decreased expression of* miR-34s* in several types of malignant cancers, the* miR-34* family powerfully prevents tumorigenesis in general.

**Figure 1:**
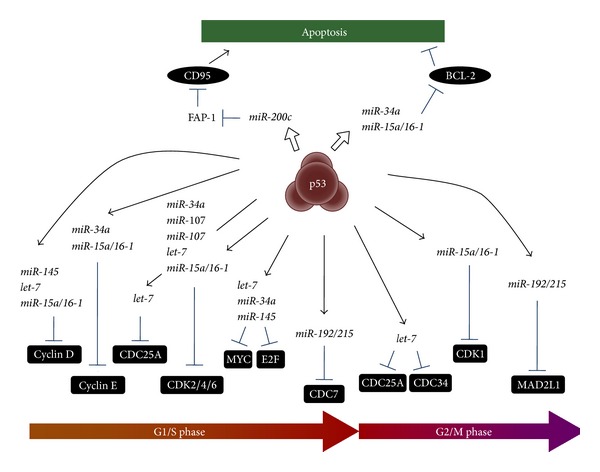
p53-induced miRNAs control cell cycle and cell survival. p53 directly induces many kinds of miRNAs, which repress cell-cycle regulators and/or antiapoptotic proteins and contribute to cell-cycle arrest and apoptosis. The miRNAs regulating apoptosis are shown in the top part of this figure, and the miRNAs regulating the cell cycle are at the bottom.

In addition to the* miR-34* family, p53 is also engaged in the direct regulation of the transcriptional expression of additional miRNAs, such as* miR-107*,* miR-143/145*,* miR-192/194/215*,* miR-200c/141*, the* let-7* family, and the* miR-17-92* cluster (Figure 1 and Table 1).

### 4.2. *miR-107*

*miR-107* is encoded within an intron of pantothenate kinase 1 (*PANK1*), and* miR-107* and its host gene are directly activated by p53 under hypoxia condition or with the treatment of DNA damage agents [55, 56]. Hypoxia induces angiogenesis, which is essential for solid tumors to grow in severe environments.* miR-107* inhibits hypoxia signaling and antiangiogenesis by repressing the expression of hypoxia inducible factor-1*β* (HIF-1*β*), which interacts with HIF-1α to form the HIF-1 transcription factor complex [55]. Furthermore,* miR-107* promotes cell-cycle arrest in the G_1_/S phase via targeting the cell-cycle activator CDK6 and the antimitogenic p130 [56]. Nevertheless,* miR-107* has another aspect for directly targeting* DICER1* mRNA and the high level of* miR-107* might affect the production and function of p53-induced miRNAs [57].

### 4.3. *miR-145*

It has been reported that the expression of* miR-145* is frequently decreased in colon tumors, breast and prostate cancers and that the chromosomal region (chromosome 5 [5q32-33] within a 4.09 kb region) is deleted in myelodysplastic syndrome, suggesting* miR-145* acts as a tumor suppressor [58–61]. The expression of* miR-145* is transcriptionally induced by p53, and* miR-145* downregulates c-MYC, E2F3, cyclin D2, CDK4, and CDK6 and leads to G_1_ cell-cycle arrest [62, 63].

Recently, it has been found that* miR-145* contains several CpG sites in its promoter region and that the expression of* miR-145* is affected by epigenetic events such as DNA methylation [60]. The CpG regions are located adjacent to p53 response element upstream of miR-145, and DNA hypermethylation inhibits p53 from binding to* miR-145*. In addition to this miRNA, it has been reported that* miR-34a*,* miR-124a*, and* miR-127* are downregulated by DNA methylation [64].

### 4.4. *miR-192/215*

*miR-192* and* miR-215* share a similar seed sequence and are composed of two clusters: the* miR-215*/*miR-194-1* cluster on chromosome 1 (1q41) and the* miR-192*/*miR-194-2* cluster on chromosome 11 (11q13.1) [65].* miR-192* and* miR-215* are downregulated in colon cancers, lung cancers, multiple myeloma, and renal cancers [66–69]. Some studies have suggested that these miRNAs are also under the control of p53 and can induce p21 expression and cell-cycle arrest in a partially p53-dependent manner [66, 70]. Gene expression analyses indicated that* miR-192* and* miR-215* target a number of transcripts that regulate DNA synthesis and the G_1_ and G_2_ cell-cycle checkpoints, such as CDC7 and MAD2L1 [70]. Therefore,* miR-192/215* functions as a tumor suppressor contributing to the G_1_ and G_2_/M cell-cycle arrest.

### 4.5. *miR-200c*

It is well known that p53 acts as an important regulator in modulating epithelial-mesenchymal transition (EMT) that is implicated in tumor progression, metastasis, and the correlation of poor patient prognosis [71, 72]. The p53-induced* miR-200c* represses EMT by targeting the E-cadherin transcriptional repressors ZEB1 and ZEB2, Krüppel-like factor 4 (KLF4), and the polycomb repressor BMI1, all of which are involved in the maintenance of stemness [73–80]. Moreover,* miR-200c* contributes to the induction of apoptosis in cancer cells via the apoptosis-inducing receptor CD95 by targeting the apoptosis-inhibitor FAS-associated phosphatase 1 (FAP-1) [81].

### 4.6. *let-7a* and* let-7b*

*let-7* is known to be important for the regulation of development and is evolutionally conserved across bilaterian phylogeny [82]. In humans, some* let-7* gene clusters are located in fragile regions involved in cancers [61]. In lung cancers, it has been reported that the downregulated expression of* let-7* members is correlated with poor prognosis [83, 84]. Recent works suggested that* let-7a* and* let-7b* expression is dependent on p53 in response to genotoxic stress and* let-7* miRNAs target CDK6, CDC25A, cyclin D, CDC34, and MYC [85–89]. On the other hand,* let-7a-d* and* let-7i* are direct targets of E2F1 and E2F3 during the G_1_/S transition and are repressed in E2F1/3-null cells [90]. The* let-7* family plays multiple roles in the regulation of the cell cycle and goes a long way toward suppressing tumor progression.

### 4.7. *miR-17-92* Cluster

The* miR-17-92* cluster consists of* miR-17-5p*,* miR-17-3p*,* miR-18a*,* miR-19a*,* miR-20a*,* miR-19b*, and* miR-92-1*. Some of these are known to be oncogenic, as suggested in the research showing that the cluster is upregulated in human B-cell lymphoma and amplified in malignant lymphoma [91, 92].

Different from the miRNAs mentioned above,* miR-17-92* miRNAs are more or less repressed transcriptionally by p53 under hypoxia, which leads to the p53-mediated apoptosis [93]. The p53-binding site overlaps with the TATA box of the* miR-17-92* promoter region, and p53 prevents the TATA-binding protein (TBP) transcription factor from binding to the site during hypoxic conditions. Moreover,* miR-17-92* is transcriptionally regulated by c-Myc [94]. Although c-Myc is repressed by p53 activation under some stress conditions, the repression of* miR-17-92* is not dependent on c-MYC but on p53 under hypoxia [93, 95].

Note that some members of* miR-17-92* are likely to function as tumor suppressors in different cancers. For example, in breast cancer,* miR-17-5p* represses the expression of the nuclear receptor coactivator amplified in breast cancer 1 (*AIB1*) that enhances the transcription activity of E2F1 to promote the cell proliferation of breast cancer cells [96]. A recent study showed that* miR-17-3p* reduces tumor growth by targeting MDM2 in glioblastoma cells [97].

### 4.8. *miR-15a/miR-16-1*

*miR-15a* and* miR-16-1* were identified to be deleted and/or downregulated in approximately 68% of B-cell chronic lymphocytic leukemia (B-CLL) [98], as is the case in pituitary adenomas [99], gastric cancer cells [100], prostate cancer [101–104], non-small cell lung cancer [105, 106], ovarian cancer [107], and pancreatic cancer [108], which indicates their important functions for tumor formation. The miRNAs are encoded by an intron of a long noncoding RNA gene, deleted in lymphocytic leukemia 2 (*DLEU2*), and* DLEU2* (*miR-15a/miR-16-1*) was shown to be transactivated by p53 [109]. In addition, p53 regulates the expression level of precursor and mature* miR-15a* and* miR-16-1* as well as* miR-143* and* miR-145* [110]. It has been reported that miR-15a/miR-16-1 negatively regulates the antiapoptotic protein BCL-2 and the cell-cycle regulators, such as CDK1, CDK2, and CDK6, and cyclins D1, D3, and E1 [102, 110–112].

## 5. miRNAs Regulating Negative Regulators of p53

**Figure 2:**
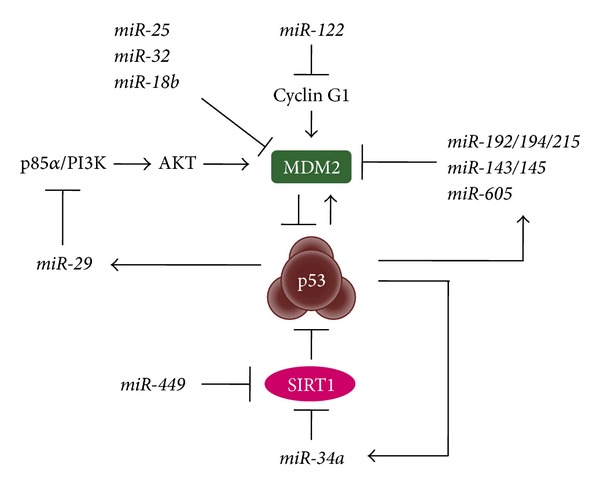
Indirect p53 regulation with miRNAs. p53 controls its stability and activity with the p53-inducible miRNAs that directly or indirectly target the negative regulators (MDM2 and SIRT1).* miR-25*,* miR-32*,* miR-18b*, and* miR-449* are not direct targets of p53 but repress the negative regulators and lead to p53 activation.

It has been shown that MDM2 negatively controls the stability and transcription activity of p53, which attenuates the tumor-suppressive functions of p53 [40]. Actually, overexpression of MDM2 is often found in many types of human cancers, such as soft tissue sarcomas, brain tumors, and head and neck squamous cell carcinomas [113, 114]. On the flip side, p53 inhibits MDM2 expression using several miRNAs and establishes the regulatory circuit between p53 and MDM2 (Figure 2). For instance,* miR-192/194/215*,* miR-143/145*, and* miR-605*, which are the transcriptional targets of p53, directly inhibit MDM2 expression [68, 114, 115].* miR-29* family members are also p53-inducible miRNAs and indirectly control the MDM2 level by targeting p85*α*, a regulatory subunit of PI3 kinase (PI3K), in the PI3K/AKT/MDM2 axis [116, 117]. Furthermore, the* miR-29* family directly suppresses cell division cycle 42 (CDC42) and PPM1D phosphatase, both of which negatively regulate p53 [116, 117]. While a liver-specific* miR-122* is not a transcriptional target of p53, the miRNA increases p53 activity through the downregulation of cyclin G1, which inhibits the recruitment of phosphatase 2A (PP2A) to dephosphorylate MDM2 and causes the decrease of MDM2 activity [118, 119]. Recent studies indicated that tumor-suppressive miRNAs,* miR-25*,* miR-32*, and* miR-18b* are also not transcriptionally regulated by p53 but affect the p53 pathway by targeting* MDM2* mRNA directly [120, 121].

Besides MDM2, a NAD-dependent deacetylase, silent information regulator 1 (SIRT1), increases the level of deacetylated p53 and negatively regulates the p53 activity [122, 123]. SIRT1 is targeted by the p53-inducible* miR-34a* and joins the positive feedback loop connecting the miRNA, SIRT1, and p53 (Figure 2) [124]. Additionally,* miR-499* participates in this regulatory circuit as the miRNA possesses a very similar seed sequence of* miR-34* members [125–127].* miR-449* is upregulated by E2F1, not by p53, and* miR-34* and* miR-449* bring in an asymmetric network to balance the functions between p53 and E2F1.

## 6. miRNAs Directly Targeting* TP53* mRNA

**Figure 3:**
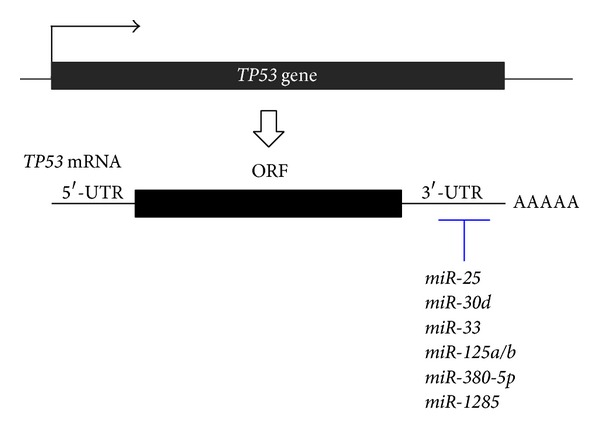
Direct p53 regulation by miRNAs. miRNAs directly interact with* TP53* mRNA by binding to sites in the 3′-UTR. This interaction inhibits the translation of mRNA, resulting in the repression of p53 activity. ORF: open reading frame.

As is the case in the control of negative regulators of p53 via miRNAs, p53 itself is repressed by several miRNAs through direct interaction with the 3′-UTR of* TP53* mRNA (Figure 3).

*miR-125b* is a first-identified p53-repressive miRNA and blocks the p53 expression level to suppress apoptosis in human neuroblastoma and lung fibroblast cells; in contrast, the knockdown of* miR-125b* leads to the opposite results [128]. Plus,* miR-125a*, an isoform of* miR-125*, was suggested to inhibit the translation of* TP53* by binding to a region of the 3′-UTR [129]. The high expression of* miR-125b* is associated with poor prognosis in patients with colorectal cancer [130]. Some studies have shown that* miR-125b* represses factors in the p53 network, including apoptosis regulators like* PUMA*, insulin-like growth factor-binding protein 3 (*IGFBP3*), and BCL2-antagonist/killer 1 (*BAK1*) and cell-cycle regulators like* cyclin C*,* CDC25C*, and cyclin-dependent kinase inhibitor 2C (*CDKN2C*) [131]. These suggest that* miR-125b* modulates and buffers the p53 pathway.

Subsequently,* miR-504* was reported to directly repress the p53 protein level and reduce the p53-mediated apoptosis and cell-cycle arrest in response to stress, and its overexpression promotes the tumorigenicity of colon cancer cells* in vivo* [132]. Additionally,* miR-380-5p*,* miR-33*, and* miR-1285* can downregulate the p53 protein expression by directly binding to the two sites in the 3′-UTR of* TP53*, resulting in the reduction of apoptosis and cell-cycle arrest [133–135]. Indeed,* miR-380-5p* is highly expressed in neuroblastomas with neuroblastoma-derived v-myc myelocytomatosis viral-related oncogene (*MYCN*) amplification, and the high expression level correlates with poor diagnosis [133]. More recently,* miR-30d* and* miR-25* also directly interacted with the 3′-UTR of* TP53* to decrease the p53 level. So then, these miRNAs affect apoptotic cell death, cell-cycle arrest, and cellular senescence in some cell lines, such as multiple myelomas, colon cancer, and lung cancer cells [136–138]. When taken together, the miRNAs targeting* TP53* would hinder p53 from exerting its tumor-suppressive functions (senescence, apoptosis, cell-cycle arrest, etc.) under stressed conditions.

## 7. Concluding Remarks

For more than a decade, small noncoding RNAs have become increasingly central to the study of tumor biology. The accumulating evidence of cancer-associated miRNAs reveals the missing link between classic tumor-suppressive networks and complex oncogenic pathways. In a stress situation, p53 directly induces various protein-coding genes such as* p21* and* PUMA* to contribute to cell-cycle arrest and apoptosis and, furthermore, utilizes tumor-suppressive miRNAs, such as* miR-34s*,* miR-107*, and* miR-145* (Figure 1 and Table 1). Some of the p53-inducible miRNAs target p53-negative regulators (MDM2 and SIRT1), which creates a positive feedback loop to reinforce p53 stability and activity (Figure 2). However, as expected, miRNAs are not always on p53's side: p53-repressive miRNAs (*miR-125s*,* miR-504*,* miR-380-5p*, etc.) reduce the p53 expression level by binding to a region of the 3′-UTR of* TP53* mRNA and result in the inhibition of cell-cycle arrest and apoptosis (Figure 3). There will be more than one way to arrest the cell-cycle and/or induce apoptosis, and the balance between miRNAs and tumor suppressors might be crucial in deciding which strategy to adapt.

For future diagnostic and therapeutic advances, more extensive studies will be needed to find hidden messages in the tumor-suppressive networks of miRNA. The regulatory mechanism of the p53-miRNA circuit has been excellently shown, but the upstream regulators of almost all miRNAs are unknown at this time. What is more, regardless of computational prediction, the downstream targets of miRNA are hard to identify exactly because of the imperfect complementarity and the possibility that miRNAs can bind to not only the 3′-UTR but also the 5′-UTR and coding regions.

In recent years, the competitive endogenous RNA (ceRNA) hypothesis has suggested that noncoding pseudogenes and long noncoding RNAs act as miRNA sponges, which is likely to counteract the effect of miRNAs on the target mRNA transcripts [139]. Therefore, we need to move deeper inside the world of noncoding RNAs in order to prevent and treat diverse cancers.

Besides the miRNAs described in this paper, there are many miRNAs related to cell-cycle regulation and apoptosis [140–142]. However, it is unclear how these miRNAs act additively/synergistically on tumor suppression. Even the longest journey to understand the role of miRNA begins with a single experiment. The next ten years will be more exciting in the quest to see cancer conquered.

## Figures and Tables

**Table 1:** Key microRNAs regulated by p53.

miRNA	Genomic location	Cancer type	Target	Phenotype	References
*miR-34s *	1p36 and 11q23	Colon cancer, neuroblastoma, pancreatic cancer, CLL, NSCLC, OSCC, breast cancer, bladder cancer, kidney cancer, melanoma	CDK4, CDK6, cyclin E2, E2F3, MYC BCL-2	Cell-cycle arrest apoptosis	[14–16, 18, 51, 53, 54]

*miR-107 *	10q23	Colon cancer, breast cancer	CDK6, P130	Cell-cycle arrest	[55–57]

*miR-145 *	5q23	Colon cancer, breast cancer, MDS, prostate cancer	MYC, E2F3, cyclin D2, CDK4, CDK6	Cell-cycle arrest	[58–61]

*miR-192/215 *	1q41 and 11q13	Colon cancer, lung cancer, multiple myeloma, renal cancer	CDC7, MAD2L1	Cell-cycle arrest	[66–69]

*miR-200c *	12p13	Breast cancer, ovarian cancer	FAP-1	Apoptosis	[71, 81]

*let-7 *	Multiple locations (11 copies)	Lung cancer, colon cancer, ovarian cancer, breast cancer, lymphoma	CDK6, CDC25A, cyclin D, CDC34, MYC, E2F1, E2F3	Cell-cycle arrest	[61, 83–90]

*miR-15a/16-1 *	13q14	B-CLL, pituitary adenomas, gastric cancer, NSCLC, prostate cancer, ovarian cancer, pancreatic cancer	CDK1, CDK2, CDK6, cyclin D1, D3, E1 BCL-2	Cell-cycle arrest apoptosis	[98–112]

CLL: chronic lymphocytic leukemia; NSCLC: non-small cell lung cancer; OSCC: oral squamous cell carcinoma; MDS: myelodysplastic syndromes; B-CLL: B-cell chronic lymphocytic leukemia.

## References

[1] Calin GA, Croce CM (2006). MicroRNA signatures in human cancers. *Nature Reviews Cancer*.

[2] Vousden KH, Lane DP (2007). p53 in health and disease. *Nature Reviews Molecular Cell Biology*.

[3] Pelengaris S, Khan M, Evan G (2002). c-MYC: more than just a matter of life and death. *Nature Reviews Cancer*.

[4] Vogelstein B, Kinzler KW (2004). Cancer genes and the pathways they control. *Nature Medicine*.

[5] Olivier M, Hussain SP, de Fromentel CC, Hainaut P, Harris CC (2004). TP53 mutation spectra and load: a tool for generating hypotheses on the etiology of cancer. *IARC Scientific Publications*.

[6] Levine AJ, Hu W, Feng Z (2006). The P53 pathway: what questions remain to be explored?. *Cell Death and Differentiation*.

[7] El-Deiry WS, Tokino T, Velculescu VE (1993). WAF1, a potential mediator of p53 tumor suppression. *Cell*.

[8] Yu J, Zhang L, Hwang PM, Kinzler KW, Vogelstein B (2001). PUMA induces the rapid apoptosis of colorectal cancer cells. *Molecular Cell*.

[9] Hermeking H, Lengauer C, Polyak K (1997). 14-3-3σ is a p53-regulated inhibitor of G2/M progression. *Molecular Cell*.

[10] Vousden KH, Prives C (2009). Blinded by the light: the growing complexity of p53. *Cell*.

[11] Spurgers KB, Gold DL, Coombes KR (2006). Identification of cell cycle regulatory genes as principal targets of p53-mediated transcriptional repression. *The Journal of Biological Chemistry*.

[12] Lu J, Getz G, Miska EA (2005). MicroRNA expression profiles classify human cancers. *Nature*.

[13] Lujambio A, Lowe SW (2012). The microcosmos of cancer. *Nature*.

[14] Raver-Shapira N, Marciano E, Meiri E (2007). Transcriptional activation of miR-34a contributes to p53-mediated apoptosis. *Molecular Cell*.

[15] Chang T-C, Wentzel EA, Kent OA (2007). Transactivation of miR-34a by p53 broadly influences gene expression and promotes apoptosis. *Molecular Cell*.

[16] He L, He X, Lim LP (2007). A microRNA component of the p53 tumour suppressor network. *Nature*.

[17] Tarasov V, Jung P, Verdoodt B (2007). Differential regulation of microRNAs by p53 revealed by massively parallel sequencing: *miR-34a* is a p53 target that induces apoptosis and G_1_-arrest. *Cell Cycle*.

[18] Bommer GT, Gerin I, Feng Y (2007). p53-mediated activation of miRNA34 candidate tumor-suppressor genes. *Current Biology*.

[19] Lee RC, Feinbaum RL, Ambros V (1993). The *C. elegans* heterochronic gene *lin-4* encodes small RNAs with antisense complementarity to *lin-14*. *Cell*.

[20] Wightman B, Ha I, Ruvkun G (1993). Posttranscriptional regulation of the heterochronic gene *lin-14* by *lin-4* mediates temporal pattern formation in *C. elegans*. *Cell*.

[21] Reinhart BJ, Slack FJ, Basson M (2000). The 21-nucleotide let-7 RNA regulates developmental timing in *Caenorhabditis elegans*. *Nature*.

[22] Lewis BP, Burge CB, Bartel DP (2005). Conserved seed pairing, often flanked by adenosines, indicates that thousands of human genes are microRNA targets. *Cell*.

[23] Krek A, Grün D, Poy MN (2005). Combinatorial microRNA target predictions. *Nature Genetics*.

[24] Lee Y, Kim M, Han JJ (2004). MicroRNA genes are transcribed by RNA polymerase II. *The EMBO Journal*.

[25] Borchert GM, Lanier W, Davidson BL (2006). RNA polymerase III transcribes human microRNAs. *Nature Structural and Molecular Biology*.

[26] Kim VN (2005). MicroRNA biogenesis: coordinated cropping and dicing. *Nature Reviews Molecular Cell Biology*.

[27] Lee Y, Jeon K, Lee J-T, Kim S, Kim VN (2002). MicroRNA maturation: stepwise processing and subcellular localization. *The EMBO Journal*.

[28] Lund E, Güttinger S, Calado A, Dahlberg JE, Kutay U (2004). Nuclear export of microRNA precursors. *Science*.

[29] Bartel DP (2004). MicroRNAs: genomics, biogenesis, mechanism, and function. *Cell*.

[30] Kim VN (2005). Small RNAs: classification, biogenesis, and function. *Molecules and Cells*.

[31] Lim LP, Lau NC, Garrett-Engele P (2005). Microarray analysis shows that some microRNAs downregulate large numbers of-target mRNAs. *Nature*.

[32] Bagga S, Bracht J, Hunter S (2005). Regulation by *let-7* and *lin-4* miRNAs results in target mRNA degradation. *Cell*.

[33] Lai EC (2004). Predicting and validating microRNA targets. *Genome Biology*.

[34] Wu L, Belasco JG (2008). Let me count the ways: mechanisms of gene regulation by miRNAs and siRNAs. *Molecular Cell*.

[35] Eulalio A, Huntzinger E, Izaurralde E (2008). Getting to the root of miRNA-mediated gene silencing. *Cell*.

[36] Carthew RW, Sontheimer EJ (2009). Origins and mechanisms of miRNAs and siRNAs. *Cell*.

[37] Yates LA, Norbury CJ, Gilbert RJC (2013). The long and short of microRNA. *Cell*.

[38] Haupt Y, Maya R, Kazaz A, Oren M (1997). Mdm2 promotes the rapid degradation of p53. *Nature*.

[39] Kubbutat MHG, Jones SN, Vousden KH (1997). Regulation of p53 stability by Mdm2. *Nature*.

[40] Brooks CL, Gu W (2006). p53 ubiquitination: mdm2 and beyond. *Molecular Cell*.

[41] Roger L, Gadea G, Roux P (2006). Control of cell migration: a tumour suppressor function for p53?. *Biology of the Cell*.

[42] Teodoro JG, Parker AE, Zhu X, Green MR (2006). p53-mediated inhibition of angiogenesis through up-regulation of a collagen prolyl hydroxylase. *Science*.

[43] Junttila MR, Evan GI (2009). P53—a Jack of all trades but master of none. *Nature Reviews Cancer*.

[44] Gatz SA, Wiesmüller L (2006). p53 in recombination and repair. *Cell Death and Differentiation*.

[45] Bensaad K, Vousden KH (2005). Savior and slayer: the two faces of p53. *Nature Medicine*.

[46] Bensaad K, Tsuruta A, Selak MA (2006). TIGAR, a p53-inducible regulator of glycolysis and apoptosis. *Cell*.

[47] Matoba S, Kang J-G, Patino WD (2006). p53 regulates mitochondrial respiration. *Science*.

[48] Murray-Zmijewski F, Lane DP, Bourdon J-C (2006). p53/p63/p73 isoforms: an orchestra of isoforms to harmonise cell differentiation and response to stress. *Cell Death and Differentiation*.

[49] Cawley S, Bekiranov S, Ng HH (2004). Unbiased mapping of transcription factor binding sites along human chromosomes 21 and 22 points to widespread regulation of noncoding RNAs. *Cell*.

[50] Wei C-L, Wu Q, Vega VB (2006). A global map of p53 transcription-factor binding sites in the human genome. *Cell*.

[51] Tazawa H, Tsuchiya N, Izumiya M, Nakagama H (2007). Tumor-suppressive *miR-34a* induces senescence-like growth arrest through modulation of the E2F pathway in human colon cancer cells. *Proceedings of the National Academy of Sciences of the United States of America*.

[52] Corney DC, Flesken-Nikitin A, Godwin AK, Wang W, Nikitin AY (2007). *MicroRNA-34b* and *MicroRNA-34c* are targets of p53 and cooperate in control of cell proliferation and adhesion-independent growth. *Cancer Research*.

[53] Hermeking H (2012). MicroRNAs in the p53 network: micromanagement of tumour suppression. *Nature Reviews Cancer*.

[54] Hermeking H (2010). The *miR-34* family in cancer and apoptosis. *Cell Death & Differentiation*.

[55] Yamakuchi M, Lotterman CD, Bao C (2010). P53-induced microRNA-107 inhibits HIF-1 and tumor angiogenesis. *Proceedings of the National Academy of Sciences of the United States of America*.

[56] Böhlig L, Friedrich M, Engeland K (2011). P53 activates the *PANK1/miRNA-107* gene leading to downregulation of CDK6 and p130 cell cycle proteins. *Nucleic Acids Research*.

[57] Martello G, Rosato A, Ferrari F (2010). A microRNA targeting dicer for metastasis control. *Cell*.

[58] Michael MZ, O'Connor SM, van Holst Pellekaan NG, Young GP, James RJ (2003). Reduced accumulation of specific microRNAs in colorectal neoplasia. *Molecular Cancer Research*.

[59] Iorio MV, Ferracin M, Liu C-G (2005). MicroRNA gene expression deregulation in human breast cancer. *Cancer Research*.

[60] Suh SO, Chen Y, Zaman MS (2011). MicroRNA-145 is regulated by DNA methylation and p53 gene mutation in prostate cancer. *Carcinogenesis*.

[61] Calin GA, Sevignani C, Dumitru CD (2004). Human microRNA genes are frequently located at fragile sites and genomic regions involved in cancers. *Proceedings of the National Academy of Sciences of the United States of America*.

[62] Sachdeva M, Zhu SM, Wu FT (2009). p53 represses c-Myc through induction of the tumor suppressor *miR-145*. *Proceedings of the National Academy of Sciences of the United States of America*.

[63] Zhu H, Dougherty U, Robinson V (2011). EGFR signals downregulate tumor suppressors miR-143 and miR-145 in Western diet-promoted murine colon cancer: role of G_1_ regulators. *Molecular Cancer Research*.

[64] Lujambio A, Esteller M (2007). CpG island hypermethylation of tumor suppressor microRNAs in human cancer. *Cell Cycle*.

[65] Khella HW, Bakhet M, Allo G (2013). miR-192, miR-194 and miR-215: a convergent microRNA network suppressing tumor progression in renal cell carcinoma. *Carcinogenesis*.

[66] Braun CJ, Zhang X, Savelyeva I (2008). p53-responsive microRNAs 192 and 215 are capable of inducing cell cycle arrest. *Cancer Research*.

[67] Feng S, Cong S, Zhang X (2011). MicroRNA-192 targeting retinoblastoma 1 inhibits cell proliferation and induces cell apoptosis in lung cancer cells. *Nucleic Acids Research*.

[68] Pichiorri F, Suh S-S, Rocci A (2010). Downregulation of p53-inducible microRNAs *192*, *194*, and *215* impairs the p53/MDM2 autoregulatory loop in multiple myeloma development. *Cancer Cell*.

[69] Senanayake U, Das S, Vesely P (2012). miR-192, miR-194, miR-215, miR-200c and miR-141 are downregulated and their common target ACVR2B is strongly expressed in renal childhood neoplasms. *Carcinogenesis*.

[70] Georges SA, Biery MC, Kim S-Y (2008). Coordinated regulation of cell cycle transcripts by p53-inducible microRNAs, miR-192 and miR-215. *Cancer Research*.

[71] Polyak K, Weinberg RA (2009). Transitions between epithelial and mesenchymal states: acquisition of malignant and stem cell traits. *Nature Reviews Cancer*.

[72] Valastyan S, Weinberg RA (2011). Tumor metastasis: molecular insights and evolving paradigms. *Cell*.

[73] Chang C, Chao CH, Xia W (2011). P53 regulates epithelial-mesenchymal transition and stem cell properties through modulating miRNAs. *Nature Cell Biology*.

[74] Kim T, Veronese A, Pichiorri F (2011). p53 regulates epithelial-mesenchymal transition through microRNAs targeting ZEB1 and ZEB2. *Journal of Experimental Medicine*.

[75] Burk U, Schubert J, Wellner U (2008). A reciprocal repression between ZEB1 and members of the miR-200 family promotes EMT and invasion in cancer cells. *EMBO Reports*.

[76] Gregory PA, Bert AG, Paterson EL (2008). The miR-200 family and miR-205 regulate epithelial to mesenchymal transition by targeting ZEB1 and SIP1. *Nature Cell Biology*.

[77] Korpal M, Lee ES, Hu G, Kang Y (2008). The miR-200 family inhibits epithelial-mesenchymal transition and cancer cell migration by direct targeting of E-cadherin transcriptional repressors *ZEB1* and *ZEB2*. *The Journal of Biological Chemistry*.

[78] Park S-M, Gaur AB, Lengyel E, Peter ME (2008). The miR-200 family determines the epithelial phenotype of cancer cells by targeting the E-cadherin repressors ZEB1 and ZEB2. *Genes & Development*.

[79] Shimono Y, Zabala M, Cho RW (2009). Downregulation of miRNA-200c links breast cancer stem cells with normal stem cells. *Cell*.

[80] Wellner U, Schubert J, Burk UC (2009). The EMT-activator ZEB1 promotes tumorigenicity by repressing stemness-inhibiting microRNAs. *Nature Cell Biology*.

[81] Schickel R, Park S-M, Murmann AE, Peter ME (2010). miR-200c regulates induction of apoptosis through CD95 by targeting FAP-1. *Molecular Cell*.

[82] Pasquinelli AE, Reinhart BJ, Slack F (2000). Conservation of the sequence and temporal expression of let-7 heterochronic regulatory RNA. *Nature*.

[83] Takamizawa J, Konishi H, Yanagisawa K (2004). Reduced expression of the let-7 microRNAs in human lung cancers in association with shortened postoperative survival. *Cancer Research*.

[84] Yanaihara N, Caplen N, Bowman E (2006). Unique microRNA molecular profiles in lung cancer diagnosis and prognosis. *Cancer Cell*.

[85] Saleh AD, Savage JE, Cao L (2011). Cellular stress induced alterations in microrna let-7a and let-7b expression are dependent on p53. *PLoS ONE*.

[86] Johnson CD, Esquela-Kerscher A, Stefani G (2007). The let-7 microRNA represses cell proliferation pathways in human cells. *Cancer Research*.

[87] Legesse-Miller A, Elemento O, Pfau SJ, Forman JJ, Tavazoie S, Coller HA (2009). *Let-7* overexpression leads to an increased fraction of cells in G_2_/M, direct down-regulation of Cdc34, and stabilization of wee1 kinase in primary fibroblasts. *The Journal of Biological Chemistry*.

[88] Sampson VB, Rong NH, Han J (2007). MicroRNA let-7a down-regulates MYC and reverts MYC-induced growth in Burkitt lymphoma cells. *Cancer Research*.

[89] Bueno MJ, Gómez de Cedrón M, Gómez-López G (2011). Combinatorial effects of microRNAs to suppress the Myc oncogenic pathway. *Blood*.

[90] Bueno MJ, de Cedrón MG, Laresgoiti U, Fernández-Piqueras J, Zubiaga AM, Malumbres M (2010). Multiple E2F-induced microRNAs prevent replicative stress in response to mitogenic signaling. *Molecular and Cellular Biology*.

[91] Ota A, Tagawa H, Karnan S (2004). Identification and characterization of a novel gene, *C13orf25* , as a target for 13q31-q32 amplification in malignant lymphoma. *Cancer Research*.

[92] He L, Thomson JM, Hemann MT (2005). A microRNA polycistron as a potential human oncogene. *Nature*.

[93] Yan H-L, Xue G, Mei Q (2009). Repression of the *miR-17-92* cluster by p53 has an important function in hypoxia-induced apoptosis. *EMBO Journal*.

[94] O'Donnell KA, Wentzel EA, Zeller KI, Dang CV, Mendell JT (2005). c-Myc-regulated microRNAs modulate E2F1 expression. *Nature*.

[95] Ho JSL, Ma W, Mao DYL, Benchimol S (2005). p53-dependent transcriptional repression of c-myc is required for G_1_ cell cycle arrest. *Molecular and Cellular Biology*.

[96] Hossain A, Kuo MT, Saunders GF (2006). *Mir-17-5p* regulates breast cancer cell proliferation by inhibiting translation of *AIB1* mRNA. *Molecular and Cellular Biology*.

[97] Li H, Yang BB (2012). Stress response of glioblastoma cells mediated by miR-17-5p targeting PTEN and the passenger strand miR-17-3p targeting MDM2. *Oncotarget*.

[98] Calin GA, Dumitru CD, Shimizu M (2002). Frequent deletions and down-regulation of micro-RNA genes *miR15* and *miR16* at 13q14 in chronic lymphocytic leukemia. *Proceedings of the National Academy of Sciences of the United States of America*.

[99] Bottoni A, Piccin D, Tagliati F, Luchin A, Zatelli MC, Uberti ECD (2005). miR-15a and miR-16-1 down-regulation in pituitary adenomas. *Journal of Cellular Physiology*.

[100] Xia L, Zhang D, Du R (2008). miR-15b and miR-16 modulate multidrug resistance by targeting BCL2 in human gastric cancer cells. *International Journal of Cancer*.

[101] Bonci D, Coppola V, Musumeci M (2008). The *miR-15a-miR-16-1* cluster controls prostate cancer by targeting multiple oncogenic activities. *Nature Medicine*.

[102] Takeshita F, Patrawala L, Osaki M (2010). Systemic delivery of synthetic microRNA-16 inhibits the growth of metastatic prostate tumors via downregulation of multiple cell-cycle genes. *Molecular Therapy*.

[103] Musumeci M, Coppola V, Addario A (2011). Control of tumor and microenvironment cross-talk by miR-15a and miR-16 in prostate cancer. *Oncogene*.

[104] Porkka KP, Ogg E-L, Saramäki OR (2011). The *miR-15a-miR-16-1* locus is homozygously deleted in a subset of prostate cancers. *Genes, Chromosomes and Cancer*.

[105] Bandi N, Zbinden S, Gugger M (2009). *miR-15a* and *miR-16* are implicated in cell cycle regulation in a Rb-dependent manner and are frequently deleted or down-regulated in non-small cell lung cancer. *Cancer Research*.

[106] Bandi N, Vassella E (2011). *MiR-34a* and *miR-15a/16* are co-regulated in non-small cell lung cancer and control cell cycle progression in a synergistic and Rb-dependent manner. *Molecular Cancer*.

[107] Bhattacharya R, Nicoloso M, Arvizo R (2009). MiR-15a and MiR-16 control Bmi-1 expression in ovarian cancer. *Cancer Research*.

[108] Zhang XJ, Ye H, Zeng CW, He B, Zhang H, Chen YQ (2010). Dysregulation of miR-15a and miR-214 in human pancreatic cancer. *Journal of Hematology & Oncology*.

[109] Fabbri M, Bottoni A, Shimizu M (2011). Association of a microRNA/TP53 feedback circuitry with pathogenesis and outcome of B-cell chronic lymphocytic leukemia. *The Journal of the American Medical Association*.

[110] Suzuki HI, Yamagata K, Sugimoto K, Iwamoto T, Kato S, Miyazono K (2009). Modulation of microRNA processing by p53. *Nature*.

[111] Linsley PS, Schelter J, Burchard J (2007). Transcripts targeted by the microRNA-16 family cooperatively regulate cell cycle progression. *Molecular and Cellular Biology*.

[112] Liu Q, Fu H, Sun F (2008). miR-16 family induces cell cycle arrest by regulating multiple cell cycle genes. *Nucleic Acids Research*.

[113] Macias E, Jin A, Deisenroth C (2010). An ARF-Independent c-MYC-activated tumor suppression pathway mediated by ribosomal protein-Mdm2 interaction. *Cancer Cell*.

[114] Zhang J, Sun Q, Zhang Z, Ge S, Han Z-G, Chen W-T (2013). Loss of *microRNA-143/145* disturbs cellular growth and apoptosis of human epithelial cancers by impairing the MDM2-p53 feedback loop. *Oncogene*.

[115] Xiao J, Lin H, Luo X, Wang Z (2011). *miR-605* joins p53 network to form a p53:*miR-605*:Mdm2 positive feedback loop in response to stress. *EMBO Journal*.

[116] Park S-Y, Lee JH, Ha M, Nam J-W, Kim VN (2009). miR-29 miRNAs activate p53 by targeting p85α and CDC42. *Nature Structural and Molecular Biology*.

[117] Ugalde AP, Ramsay AJ, de la Rosa J (2011). Aging and chronic DNA damage response activate a regulatory pathway involving miR-29 and p53. *EMBO Journal*.

[118] Okamoto K, Li H, Jensen MR (2002). Cyclin G recruits PP2A to dephosphorylate Mdm2. *Molecular Cell*.

[119] Fornari F, Gramantieri L, Giovannini C (2009). MiR-122/cyclin G1 interaction modulates p53 activity and affects doxorubicin sensitivity of human hepatocarcinoma cells. *Cancer Research*.

[120] Suh S-S, Yoo JY, Nuovo GJ (2012). MicroRNAs/TP53 feedback circuitry in glioblastoma multiforme. *Proceedings of the National Academy of Sciences of the United States of America*.

[121] Dar AA, Majid S, Rittsteuer C (2013). The role of miR-18b in MDM2-p53 pathway signaling and melanoma progression. *Journal of the National Cancer Institute*.

[122] Luo J, Nikolaev AY, Imai S-I (2001). Negative control of p53 by Sir2α promotes cell survival under stress. *Cell*.

[123] Vaziri H, Dessain SK, Eaton EN (2001). *hSIR2*
^SIRT1^ functions as an NAD-dependent p53 deacetylase. *Cell*.

[124] Yamakuchi M, Ferlito M, Lowenstein CJ (2008). miR-34a repression of SIRT1 regulates apoptosis. *Proceedings of the National Academy of Sciences of the United States of America*.

[125] Lizé M, Pilarski S, Dobbelstein M (2010). E2F1-inducible microRNA 449a/b suppresses cell proliferation and promotes apoptosis. *Cell Death & Differentiation*.

[126] Bou Kheir T, Futoma-Kazmierczak E, Jacobsen A (2011). miR-449 inhibits cell proliferation and is down-regulated in gastric cancer. *Molecular Cancer*.

[127] Lizé M, Klimke A, Dobbelstein M (2011). MicroRNA-449 in cell fate determination. *Cell Cycle*.

[128] Le MTN, Teh. C, Shyh-Chang N (2009). MicroRNA-125b is a novel negative regulator of p53. *Genes and Development*.

[129] Zhang Y, Gao J-S, Tang X (2009). MicroRNA 125a and its regulation of the p53 tumor suppressor gene. *FEBS Letters*.

[130] Nishida N, Yokobori T, Mimori K (2011). MicroRNA *miR-125b* is a prognostic marker in human colorectal cancer. *International Journal of Oncology*.

[131] Le MTN, Shyh-Chang N, Khaw SL (2011). Conserved regulation of p53 network dosage by microRNA-125b occurs through evolving miRNA-target gene pairs. *PLoS Genetics*.

[132] Hu W, Chan CS, Wu R (2010). Negative regulation of tumor suppressor p53 by microRNA miR-504. *Molecular Cell*.

[133] Swarbrick A, Woods SL, Shaw A (2010). MiR-380-5p represses p53 to control cellular survival and is associated with poor outcome in *MYCN*-amplified neuroblastoma. *Nature Medicine*.

[134] Herrera-Merchan A, Cerrato C, Luengo G (2010). miR-33-mediated downregulation of p53 controls hematopoietic stem cell self-renewal. *Cell Cycle*.

[135] Tian S, Huang S, Wu S, Guo W, Li J, He X (2010). MicroRNA-1285 inhibits the expression of p53 by directly targeting its 3′ untranslated region. *Biochemical and Biophysical Research Communications*.

[136] Li J, Donath S, Li Y, Qin D, Prabhakar BS, Li P (2010). miR-30 regulates mitochondrial fission through targeting p53 and the dynamin-related protein-1 pathway. *PLoS Genetics*.

[137] Kumar M, Lu Z, Takwi AAL (2011). Negative regulation of the tumor suppressor *p53* gene by microRNAs. *Oncogene*.

[138] Li N, Kaur S, Greshock J (2012). A combined array-based comparative genomic hybridization and functional library screening approach identifies mir-30d as an oncomir in cancer. *Cancer Research*.

[139] Salmena L, Poliseno L, Tay Y, Kats L, Pandolfi PP (2011). A *ceRNA* hypothesis: the rosetta stone of a hidden RNA language?. *Cell*.

[140] Schickel R, Boyerinas B, Park S-M, Peter ME (2008). MicroRNAs: key players in the immune system, differentiation, tumorigenesis and cell death. *Oncogene*.

[141] Tsuchiya N, Izumiya M, Ogata-Kawata H (2011). Tumor suppressor *miR-22* determines p53-dependent cellular fate through post-transcriptional regulation of p21. *Cancer Research*.

[142] Bueno MJ, Malumbres M (2011). MicroRNAs and the cell cycle. *Biochimica et Biophysica Acta*.

